# Non-Invasive Serum Markers of Non-Alcoholic Fatty Liver Disease Fibrosis: Potential Tools for Detecting Patients with Cardiovascular Disease

**DOI:** 10.31083/j.rcm2509344

**Published:** 2024-09-24

**Authors:** Ling-Zi Chen, Xu-Bin Jing, Xiang Chen, Yan-Chun Xie, Yun Chen, Xian-Bin Cai

**Affiliations:** ^1^Department of Gastroenterology, The First Affiliated Hospital of Shantou University Medical College, 515041 Shantou, Guangdong, China; ^2^Department of Endoscopy Center, Cancer Hospital of Shantou University Medical College, 515041 Shantou, Guangdong, China

**Keywords:** biomarkers, cardiovascular disease, fibrosis, non-alcoholic fatty liver disease, non-alcoholic steatohepatitis

## Abstract

Non-alcoholic fatty liver disease (NAFLD), one of the most common chronic liver diseases with a prevalence of 23%–25% globally, is an independent risk factor for cardiovascular diseases (CVDs). Growing evidence indicates that the development of NAFLD, ranging from non-alcoholic fatty liver (NAFL), non-alcoholic steatohepatitis (NASH), advanced fibrosis to cirrhosis, and even hepatocellular carcinoma, is at substantial risk for CVDs, which clinically contribute to increased cardiovascular morbidity and mortality. Non-invasive serum markers assessing liver fibrosis, such as fibrosis-4 (FIB-4) score, aspartate transaminase-to-platelet ratio index (APRI), and NAFLD fibrosis score (NFS), are expected to be useful tools for clinical management of patients with CVDs. This review aims to provide an overview of the evidence for the relationship between the progression of NAFLD and CVDs and the clinical application of non-invasive markers of liver fibrosis in managing patients with CVDs.

## 1. Introduction

Non-alcoholic fatty liver disease (NAFLD) is replacing viral hepatitis as the most common chronic liver disease, with a global prevalence of 23–25% among adults. The prevalence of NAFLD varies from region to region, with the highest in the Middle East (32%) and the lowest in Africa (13%) [1]. Despite its rising burden on global public health and the economy, minimal attention has been focused on NAFLD. The prevalence of young NAFLD was augmented from 19.34 million in 1990 to 29.49 million in 2017 [2]. It was estimated that more than USD 100 billion in annual direct medical costs in the U.S. [3]. Another assessment model for NAFLD disease progression in 8 countries suggested that China had the greatest overall and relative growth in NAFLD prevalence, with up to 314 million NAFLD cases predicted by 2030 [4]. NAFLD is now considered a multisystem disease rather than a liver disease, which encompasses a spectrum of histological conditions ranging from liver steatosis non-alcoholic steatohepatitis (NASH) to liver fibrosis, increasing the prevalence of liver-related and extrahepatic complications. Moreover, a large amount of evidence has shown that NAFLD may be closely related to cardiovascular diseases (CVDs), such as atrial fibrillation [5], heart valve calcification [6], coronary artery disease [7], and heart failure [8, 9], independently of other well-known cardiovascular risk factors. Previous research has highlighted that people with NASH tend to be at a greater risk of CVDs than those with non-alcoholic fatty liver (NAFL) [10], which means the risk of CVDs might parallel the severity of NAFLD. Thus, early monitoring and identification of liver fibrosis in NAFLD patients may reduce the incidence of major adverse cardiovascular events (MACEs). Hence, non-invasive serum markers, such as fibrosis-4 index (FIB-4), aspartate transaminase-to-platelet ratio index (APRI), and NAFLD fibrosis score (NFS), which are now advocated in current guidelines to detect fibrosis, might be potential tools for CVD management. This review mainly focuses on the evidence for a relationship between the progression of NAFLD and CVDs and the clinical application of non-invasive markers of liver fibrosis in the management of patients with CVDs.

## 2. Pathophysiological Mechanism Linking NAFLD to CVDs

**Fig. 1.**
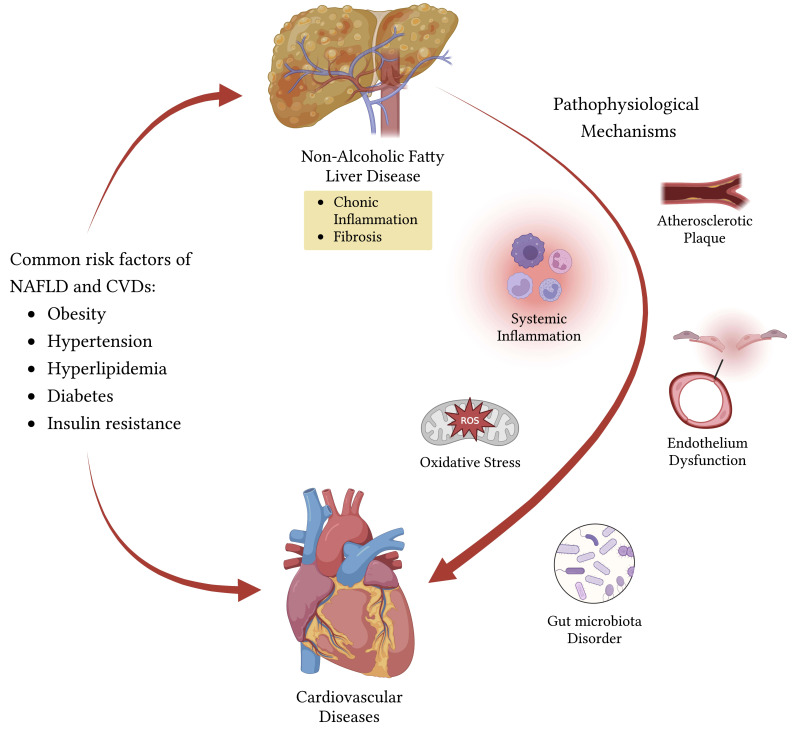
**Potential pathophysiological mechanism linking NAFLD to CVDs**. NAFLD and CVDs share common risk factors: obesity, hypertension, hyperlipidemia, diabetes, and insulin resistance. NAFLD drives multiple mechanisms that ultimately lead to CVDs, such as insulin resistance, oxidative stress, low-grade systemic inflammation, endothelial dysfunction, and gut dysbacteriosis. NAFLD, non-alcoholic fatty liver disease; CVDs, cardiovascular diseases; ROS, reactive oxygen species.

NAFLD and CVDs are both manifestations of metabolic syndrome, sharing common risk factors such as obesity, hypertension, hyperlipidemia, diabetes, and insulin resistance. The underlying mechanisms linking NAFLD to CVDs are still being researched, yet involve several complex pathways, such as insulin resistance, oxidative stress, low-grade systemic inflammation, endothelial dysfunction, and gut dysbacteriosis, which may be influenced by genetic and epigenetic variations [11] (Fig. 1). Low-grade inflammation is a key feature in the underlying mechanism between NAFLD and CVDs. Systemic inflammation promotes the occurrence of CVDs via endothelial dysfunction, enhanced plaque formation, and coagulation [12]. The vascular endothelium is involved in regulating various physiological and pathophysiological processes, such as platelet function, vascular tone, and inflammation. Recent research has indicated that impaired endothelial function plays a significant role in the interplay between NAFLD and CVDs [13, 14]. Insulin resistance would promote lipolysis in adipose tissue and increase the delivery of free fatty acids (FFAs) to the liver. FFAs induce inflammation and the production of very low-density lipoprotein (VLDL), which increases the concentration of VLDL in the circulation and leads to atherosclerosis [15, 16]. Early animal experiments demonstrated that the gut microbiota controls metabolic functions and is crucial for developing NAFLD [17]. Intestinal dysbiosis can also be involved in the development of CVDs. The gut microbiome secretes several molecules into the bloodstream; for example, many studies have suggested that trimethylamine-N-Oxide (TMAO) was associated with CVDs and considered a pro-atherogenic compound [18, 19, 20]. Emerging evidence suggested that NAFLD has been linked to CVDs, raising concerns about the early intervention of liver fibrosis in NAFLD patients with cardiovascular disease. A growing number of studies have discussed the relationship between NAFLD progression and CVDs, raising public attention to early intervention of liver fibrosis in patients with CVDs. A meta-analysis demonstrated that the fibrosis stage, determined by biopsy, was related to all-cause mortality and morbidity in patients with NAFLD, with and without adjustments for potential confounding factors [21], also providing a similar conclusion to another meta-analysis from Dulai *et al*. [22].

## 3. Progression of NAFLD and Cardiovascular Disease

NAFL is characterized as triglyceride accumulation in more than 5% of hepatocytes without evidence of hepatocellular injury or fibrosis [23]. Fat accumulation in liver cells is a key point in the development of NAFLD, further progressing to irreversible damage such as NASH and even cirrhosis under the persistence of risk factors. Hepatic triglycerides are not directly hepatotoxic, and it is estimated that hepatocyte injury is caused by toxic triglyceride precursors or triglyceride metabolites [24]. Owing to its significant relationship with metabolic syndrome, hepatic steatosis is more common in patients with obesity and hyperlipidemia, which also serve as independent risk factors for CVDs. However, individuals of normal weight (<25 kg/m^2^ in Caucasian people and <23 kg/m^2^ in Asian people) who are defined as lean and non-obese NAFLD exhibit similar, even higher cardiovascular-related mortality compared to those with obesity [25, 26]. A longitudinal, observational study from the Framingham Heart Study Third Generation cohort, over a 6.2-year follow-up period, indicated that increasing liver fat was associated with the incidence of multiple CVD risk factors, whose relationship remained significant after adjustment of baseline and changes in body mass index (BMI) [27]. Weight loss of approximately 5%–7% can decrease hepatic steatosis [28]. Bariatric and weight loss surgery has been demonstrated to improve hepatic steatosis and may be an indirect benefit to patients at high cardiovascular risk. However, few studies focus on the impact of improvement of hepatic steatosis on cardiovascular prognosis in NAFLD patients, which should be further investigated and validated in long-term follow-up studies.

Characterized by not only ≥5% hepatic steatosis but also hepatic inflammation and injury with or without fibrosis [28], NASH is a dynamic condition that could regress to simple steatosis or cause progressive liver fibrosis. Approximately 25% of NAFLD cases develop NASH, which is the second leading cause of liver transplants in the United States [29, 30]. “Multiple parallel hits” theories, including genetic factors, insulin resistance, and gut microbiota, have been put forward in the progression of NASH, among which oxidative stress is considered a critical contributor to the progression from hepatic steatosis to NASH [31]. Several observational data have demonstrated the association between NAFLD and the development of CVDs, including subclinical atherosclerosis [32], carotid atherosclerosis (CA) [33], subclinical myocardial infarction [34] (MI), or stroke [35] (Table 1, Ref. [7, 36, 37, 38, 39, 40, 41, 42, 43, 44, 45, 46, 47]).

**Table 1.** **Characteristics of studies on association between NAFLD and CVDs**.

Authors	Region	Total cases	Diagnosed method		Outcomes	OR (95% CI)	HR (95% CI)	RR
			NAFLD/MAFLD	CVDs				
Agaç MT* et al*. [36]	USA	3976	CT	CT	CAC	1.37 (1.11–1.68)	-	-
Fudim M *et al*. [37]	USA	870,535	Database record	ICD-9/10	HF	-	1.23 (1.19–1.29)	1.30
Gummesson A *et al*. [38]	Sweden	1015	CT	Ultrasound	CAC	1.77 (1.07–2.94)	-	-
Guo Y *et al*. [39]	China	11,444	Multiple criteria	ICD-10	CVDs	-	1.37 (1.20–1.56)	2.04
Kang MK *et al*. [40]	Korea	772	Ultrasound	CT	CA	1.48 (1.05–2.08)	-	1.49
Lee H *et al*. [41]	Korea	8,962,813	FLI	ICD-10	CVDs	-	NAFLD: 1.09 (1.03–1.15)	2.29
							MAFLD: 1.43 (1.41–1.45)	
Lee SB *et al*. [7]	Korea	5121	Ultrasound	CT	CA	1.18 (1.03–1.35)	-	1.32
Roh JH *et al*. [42]	Korea	308,578	FLI	ICD-10	HF	-	2.71 (2.380–3.085)	1.62
Simon TG *et al*. [43]	Sweden	56,939	Biopsy	ICD-10	MACEs	-	1.63 (1.56–1.70)	-
VanWagner LB *et al*. [44]	USA	2424	CT	CT	CAC	1.33 (1.001–1.82)	-	1.46
Wong VW *et al*. [45]	China	612	Ultrasound	CC	CAD	2.31 (1.46–3.64)	-	1.32
Yu MM *et al*. [46]	China	1683	CT	CT	MACEs	-	1.63 (1.28–2.06)	1.99
Chung GE *et al*. [47]	Korea	3300	Ultrasound	Echocardiography	LV diastolic dysfunction	1.29 (1.07–1.60)	-	-

NAFLD, non-alcoholic fatty liver disease; MAFLD, metabolic associated fatty liver disease; CVDs, cardiovascular diseases; HF, heart failure; MACEs, major adverse cardiovascular events; FLI, fatty liver index; CT, computed tomography; CA, coronary atherosclerotic; CAC, coronary artery calcium; CAD, coronary artery disease; ICD, the International Statistical Classification of Diseases and Related Health Problems; CC, cardiac catheterization; LV, left ventricular; OR, odds ratio; HR, hazard ratio; CI, confidence interval; RR, relative risk (RR was calculated by the extracted data from the research).

A nationwide cohort of Swedish adults with biopsy-confirmed NAFLD performed by Simon* et al*. [43] demonstrated that rates of fatal and non-fatal MACEs outcomes, including ischemic heart disease (IHD), congestive heart failure (CHF) and cardiovascular mortality, were significantly higher in NAFLD patients than those without. Further, a significant risk was found across all stages of NAFLD and increased with the progression of NAFLD. Compared with patients with simple steatosis, those with non-cirrhotic fibrosis and cirrhosis had significantly elevated rates of MACE outcomes (4.1/1000 and 20.2/1000 person-years, respectively). Despite this, data remain limited regarding the relationship between CVDs and different stages of NAFLD, probably because of the difficulty in specifically identifying NAFL, NASH, and stage fibrosis in the absence of liver biopsy. Hence, non-invasive assessments for advanced fibrosis, such as non-invasive serum markers, FibroScan, and magnetic resonance elastography (MRE), have gradually been used in clinical practices. An increasing number of studies investigated the relationship between CVDs and NAFLD fibrosis assessed using non-invasive serum tests, most of which concluded that the liver fibrosis stage was associated with a high risk of cardiovascular events [48, 49, 50].

## 4. Clinical Application of Non-Invasive Tests

The assessment of the NAFLD fibrosis stage plays an essential role in evaluating the prognosis, establishing therapies, and evaluating the response to treatments. Liver biopsy, the golden standard for identifying fibrosis, provides direct measurement and exact stages of hepatic fibrosis. However, due to its invasiveness, it also has well-known limitations, such as poor acceptability, sample error, and potential complications, including pain, infection, and bleeding. Thus, it seems impractical and challenging to conduct liver biopsy in large-scale clinical screening for NAFLD. Therefore, several accurate, repeatable, dynamic, and non-invasive methods have been developed in clinical practice, including imaging techniques and serum markers.

Ultrasonography is the most common imaging method for diagnosing liver steatosis owing to its low cost and easy operation, which is widely used in screening and health check-ups. However, ultrasonography can only detect moderate-to-severe hepatic steatosis (>30% liver fat) with low sensitivity for mild steatosis (<30% liver fat) [51]. Significantly, NAFLD is defined as more than 5% liver steatosis, meaning a relevant number of patients with 5%–30% liver fat might be missed using B-mode ultrasonography. In addition, the accuracy of ultrasonography for fatty liver is reduced in patients with obesity [52]. Conventional ultrasonography is qualitative and subjective, and the degree of hepatic steatosis can be scored as mild, moderate, and severe, with a poor interobserver agreement. On this basis, FibroScan, a new quantitative ultrasound-based technique, has been commonly used by hepatologists in Europe and Asia. This new technique can assess liver fat through a controlled attenuation parameter (CAP) and simultaneously obtain a liver stiffness measurement (LSM) by vibration-controlled transient elastography (VCTE). CAP and LSM are promising techniques for rapid and standardized detection of steatosis and fibrosis. However, they cannot yet be recommended as first-line measurements due to limited availability. Given that VCTE cannot reliably distinguish the histologic features of NASH, VCTE can only determine the stage of fibrosis or the presence of cirrhosis instead of diagnosing or ruling out NASH [53]. Notably, optimal CAP cut-off values for the presence or severity of steatosis are not yet defined owing to conflicting results in recent literature with different “golden standards” [54, 55]. Caussy *et al*. [54] argued that CAP-assisted detection of liver steatosis was optimized when the interquartile range (IQR) of CAP is <30 dB/m when using magnetic resonance imaging (MRI) as a gold standard [56]. Furthermore, LSM can overestimate fibrosis in case of acute hepatitis, extrahepatic cholestasis, liver congestion, and food intake. Whether the measurements of LSM are affected by M and XL probes remains unknown. For instance, the XL probe may generate a lower LSM than the M probe [57]. Although a lesser degree than the M probe, the reliability of the XL probe still decreases for patients with a BMI >30 kg/m^2^ [57]. The Rio de Janeiro Cohort Study of individuals with NAFLD and type 2 diabetes demonstrated that an increasing LSM was a risk marker for total cardiovascular events (CVEs) (HR 1.05, 95% CI: 1.01–1.08) and all-cause mortality (HR 1.04, 95% CI: 1.01–1.07), whereas an increasing CAP was a protective factor (HR 0.93, 95% CI: 0.89–0.98; HR 0.92, 95% CI: 0.88–0.97) [58]; probably because liver steatosis decreased as liver fibrosis increased. Despite this, there is less evidence about prediction ability and cut-off values of LSM and CAP for detecting high cardiovascular risk.

MRE is a MRI-based method for quantitatively imaging tissue stiffness, which appears more accurate than sonographic elastography and is not significantly impacted by obesity with a lower risk of failure [59]. A meta-analysis evaluating the diagnostic accuracy of elastography and magnetic resonance imaging for liver fibrosis and NASH demonstrated that areas under the receiver operating characteristic curve (AUROC) of MRE for diagnosis of significant fibrosis, advanced fibrosis, and cirrhosis were all above 0.90 [60]. Proton density fat fraction (PDFF) is the ratio of proton density in free triglyceride to the total proton density in free triglyceride and water, while MRI-PDFF uses different resonance frequencies of water and fat protons to determine the proportion of total hepatic protons bound to fat. The AUROCs for identifying steatosis grades 1, 2, and 3 were 0.99, 0.90, and 0.92, respectively, for MRI-PDFF, which was superior to CAP for quantifying liver steatosis [61]. Furthermore, MRI-PDFF can detect small changes exactly for liver steatosis over time [62]. However, these MRI-based techniques have several limitations, such as an impossibility in the case of coronary artery metal stents, high cost, time-consuming, and limited availability, which are more suitable for research purposes than for clinical practice. Studies were too few to estimate the relationship between the NAFLD severity measured by MRE or MRI-PDFF and CVDs. Because of these above limitations, predicting cardiovascular risk in NAFLD patients measured by MRE or MRI-PDFF is difficult and impractical.

Non-invasive methods include the above imaging measurement and the quantification of biomarkers in serum samples. Current serum biomarkers include models for diagnosing hepatic steatosis (*e.g.*, fatty liver index), grading fibrosis (*e.g.*, NFS), and direct measurements for fibrosis, such as procollagen-Ⅲ N-terminal peptide (PⅢNP). Among these, some are specific for NAFLD (BARD score (body mass index, aspartate aminotransferase-to-alanine aminotransferase ratio, diabetes score) and NFS), whereas some are now suitable for NAFLD patients, such as APRI and FIB-4, initially designed for hepatitis C. These non-invasive scoring systems perform with high negative predictive values (NPVs) but poor positive predictive values (PPVs), suggesting that they might be applied to exclude advanced fibrosis [63, 64, 65]. Non-invasive serum markers are suitable as first-line tools in primary healthcare settings to exclude advanced fibrosis, whereas MRE and FibroScan are more suitable for selecting patients who require liver biopsy in specialized hospitals. Unlike imaging methods and liver biopsy, non-invasive serum markers fulfill the requirements of an optimal method that is low-cost, available, repeatable, and dynamic, which is why non-invasive biomarkers are becoming the ideal surrogate markers for identifying advanced fibrosis.

As a common pathogenesis mechanism of NAFLD and coronary artery disease (CAD), oxidative stress has gradually been promoted to a position that cannot be ignored. Several reports have shown that enhanced oxidative stress correlates with coronary artery disease. Previous study has shown that 8-iso-prostaglandin (PG) F2alpha, a specific class of isoprostanes produced from arachidonic acid, might be the most valid marker to assess endogenous oxidative stress [66]. Recent research predicted the incidence and progression of cardiovascular disease by measuring urine or serum oxidative metabolite. For example, Schwedhelm *et al*. [67] introduced urinary 8-iso-PG F2alpha as a novel marker in addition to known risk factors of coronary heart disease. It was identified as an independent and cumulative risk marker of coronary heart disease, together with diabetes, hypertension, hypercholesterolemia, and elevated C-reactive protein (CRP). Beyond that, plasma levels of 8-iso-prostaglandinF2α (8-iso-PGF2α) were also positively correlated with coronary artery stenosis [68]. Similar results were also found, whereby plasma 8-iso-PGF2α levels were significantly elevated in acute myocardial infarction (AMI) patients compared to patients with stable and non-significant CAD [69]. In reality, elevated levels of serum soluble NOX2 (NADPH (nicotinamide adenine dinucleotide phosphate) oxidase 2)-derived peptide and urinary 8-iso-PG F2α have also been found in NAFLD patients [70]. Based on the hypothesis that oxidative stress may be involved in the common pathogenesis of NAFLD and CVD, the measurement of oxidative stress biomarkers may become a new trend to predict the risk of CVD in NAFLD patients in the future.

## 5. Non-Invasive Fibrosis Serum Markers in Detecting CVD Risks

EASL-EASDEASO Clinical Practice Guidelines recommend APRI, NFS, and FIB-4 as part of the diagnostic regimen for ruling out advanced fibrosis and further recommend these serum biomarkers to stratify the risk of liver-related outcomes in NAFLD [71]. Recent studies have further observed that the severity of liver fibrosis assessed by non-invasive scoring systems is associated with the increased risk of liver mortality and cardiac-related outcomes (Table 2, Ref. [7, 8, 48, 49, 72, 73, 74, 75, 76, 77, 78, 79, 80]).

**Table 2.** **Characteristics of studies on the association between non-invasive serum biomarkers of NAFLD and CVDs**.

Authors	Region	Total cases	Non-invasive methods	Outcomes	OR (95% CI)	HR (95% CI)
Chen Q *et al*. [49]	China	3265	NFS, FIB-4, APRI, GPR, Forns score	Cardiovascular mortality	-	NFS 3.02 (2.05–4.45)
						FIB-4 3.34 (2.29–4.86)
						APRI 1.99 (1.40–2.83)
						GPR 1.80 (1.36–2.39)
						Forns score 2.43 (1.28–4.61)
Lee SB *et al*. [7]	Korea	5121	NFS, FLI	Coronary atherosclerotic plaques	NFS 1.20 (1.08–1.42)	-
					FLI 1.37 (1.14–1.65)	
Niederseer D *et al*. [72]	Switzerland	1956	NFS	Framingham risk score	1.30 (1.09–1.54)	-
Ishiba H *et al*. [73]	Japan	366	FIB-4	CAC score	3.34 (1.16–9.85)	-
Lee J *et al*. [48]	Korea	1173	FIB-4, NFS	CAC score	FIB-4 1.70 (1.12–2.58)	-
					NFS 1.57 (1.02–2.44)	
Song DS *et al*. [74]	Korea	665	FIB-4, NFS, APRI, Forns score	CAC score	FIB-4 2.573 (1.147–5.769)	-
					NFS 3.91 (1.339–11.416)	
					APRI 2.151 (1.093–4.231)	
					Forns score 1.536 (0.698–3.383)	
Kim D *et al*. [75]	USA	11,154	FIB-4, NFS, APRI	CVD	-	High APRI 2.53 (1.33–4.83)
						High NFS 3.46 (1.91–6.25)
						High FIB-4 2.68 (1.44–4.99)
Kim JH *et al*. [76]	Korea	3,011,588	Fatty liver index	MI	-	2.16 (2.01–2.31)
Baratta F *et al*. [77]	Italy	898	NFS, FIB-4	CVD	-	NFS 2.29 (1.17–4.47)
						FIB-4 4.57 (1.61–12.98)
Lee CH *et al*. [78]	Korea	3,003,068	FLI	MI	-	1 FLI points 1.21 (1.14,1.29)
						2 FLI points 1.26 (1.17,1.35)
						3 FLI points 1.22 (1.13,1.32)
						4 FLI points 1.30 (1.21,1.40)
Chung GE *et al*. [79]	Korea	5,324,410	FLI	MI	-	FLI 30–59 vs. FLI <30 1.28 (1.22–1.34)
						FLI ≥60 vs. FLI <30 1.73 (1.63–1.84)
Park J* et al*. [8]	Korea	786,184	BARD score	HF	-	Incident HF 1.12 (1.04–1.20)
						Hospitalized HF 1.20 (1.07–1.35)
Han B *et al*. [80]	Korea	7,958,538	FLI	HF	-	FLI 30–60 vs. FLI <30 1.12 (1.08–1.17)
						FLI ≥60 vs. FLI <30 1.49 (1.41–1.58)

NFS, non-alcoholic fatty liver score; FIB-4, fibrosis-4 score; ARPI, aspartate transaminase-to-platelet ratio index; GPR, gamma-glutamyltransferase-to-platelet ratio; FLI, fatty liver index; CAC, coronary artery calcium; MI, myocardial infarction; HF, heart failure; OR, odds ratio; HR, hazard ratio; CI, confidence interval; NAFLD, non-alcoholic fatty liver disease; CVDs, cardiovascular diseases; BARD score, body mass index, aspartate aminotransferase-to-alanine aminotransferase ratio, diabetes score.

Advanced liver fibrosis stage, assessed by NFS and FIB-4, was associated with a high risk of coronary artery calcification (CAC) progression in NAFLD patients [48]. A prospective observational study over a median follow-up time of 41.4 months demonstrated that NAFLD patients with liver fibrosis identified by FIB-4 and NFS had a 4-fold increase in cardiovascular risk [77]. Another observational study involving 12,380 NAFLD patients concluded that patients with a FIB-4 score ≥2.67 had increased risks of MACEs and cardiovascular mortality, whereas NFS and APRI were insufficient to predict CVD risks [81]. During a median follow-up of 7 years, the degree of coronary stenosis was significantly greater in higher NFS categories, whereas FIB-4 was positively associated with the Gensini score and the number of diseased vessels [82]. Similar findings in our research show that both FIB-4 and APRI were significantly associated with the Gensini score and increased in higher APRI and FIB-4 categories [83].

Unfortunately, the optimal cut-offs for non-invasive serum biomarkers in ruling out (*e.g.*, FIB-4 <1.3, NFS <–1.455) and diagnosing advanced fibrosis (*e.g.*, FIB-4 >2.67, NFS >0.672) have been recognized, whereas the “gray areas” (*e.g.*, 1.3 < FIB-4 < 2.67, –1.455 < NFS < 0.672) have yet to be defined even though some research has defined them as moderate-to-severe fibrosis [84, 85]. Despite this, the exact recognition of advanced fibrosis seems more important in identifying NAFLD patients with high cardiovascular risks. Even though the current studies have investigated the relationship between cardiovascular disease and advanced fibrosis, as assessed by known cut-off values, further research is needed to explore the optimal cut-offs for identifying CVD risk. A common belief is that NASH is a steadily progressive disorder resulting in advanced fibrosis and even cirrhosis. Nevertheless, the natural course of NAFLD is dynamic, and the regression of NASH to NAFL was associated with improved advanced fibrosis [86]. Therefore, identifying serum biomarkers that may detect improvement in patients with fibrosis is a priority. The dynamic response of serum biomarkers to histological changes in NAFLD is still being studied. A recent study involving 261 NASH patients showed that changes in NFS, APRI, FIB-4, and aspartate transaminase/alanine aminotransferase (AST/ALT) ratio yielded low diagnostic accuracy for changes in liver fibrosis after 1 year of lifestyle intervention, whereas a simple panel consisting of glycosylated hemoglobin (HbA1c), platelet, and ALT normalization discriminated patients with fibrosis improvement better than the former [87]. Moreover, reductions in APRI and FIB-4 have also been significantly correlated with ≥1-stage improvement in histologic fibrosis after receiving obeticholic acid [88].

According to the current research, NAFLD patients with high fibrosis scores should be considered at high risk of developing CVDs. Early intervention, such as lifestyle modifications and drug therapy, can reverse the pathological state of NAFLD and reduce cardiovascular risk. Non-invasive serum biomarkers seem to be the optimal method for detecting cardiovascular risks, stratification, and evaluating therapeutic response after interventions. However, lacking routine screening for cardiovascular risks among NAFLD patients with advanced fibrosis might be the barrier. Integrating hepatic fibrosis screening for CVD risk stratification with the application of non-invasive biomarkers seems feasible but warrants further research and analysis. The next important step is to figure out highly sensitive and specific biomarkers that identify patients at high risk of cardiovascular events, monitor disease progression, and evaluate therapeutic response after intervention. A large number of research studies ought to investigate further whether different thresholds of fibrosis markers should be implemented in different cardiovascular diseases. The aim of this review was not to prove that imaging methods can be replaced by non-invasive biomarkers to stratify NAFLD progression but rather to highlight that non-invasive tests, as easily accessible, inexpensive, and repeatable clinical assessments, can be beneficial for physicians to identify NAFLD patients with high cardiovascular risks. Once these fibrosis scores increase, further cardiovascular risk stratification can be conducted to determine whether appropriate treatment is needed. Overall, emerging evidence emphasizes the added prognostic value of non-invasive serum markers regarding CVDs in patients with NAFLD, which have clinical implications regarding the need for CVD screening, risk stratification, and intervention in NAFLD patients with increased serum biomarkers. Further cohort studies should focus on whether improving advanced fibrosis assessed by serum biomarkers can reduce cardiovascular risks.

Among the several fibrosis biomarkers, the specific marker that is more effective in assessing NAFLD fibrosis has yet to be determined. Sun *et al*. [89] demonstrated that the FIB-4 index with a 1.30 cut-off has better diagnostic accuracy than the FIB-4 index with a 3.25 cut-off, NFS, and BARD score. However, a retrospective, multicenter cohort study of 320 patients suggested that the NAFLD fibrosis that score appears to be the best indicator of patients at cardiovascular risk [90]. Moreover, some researchers recommend diagnostic accuracy can be improved by combining serum biomarkers or even constructing a combined model of serum markers and imaging, which has a higher predictive efficiency [91].

## 6. Limitations 

Regrettably, the relationship between sarcopenia, NAFLD, and CVD is not mentioned in the review, firstly because of the lack of a generally accepted definition and the difficulty in adopting common diagnostic criteria. Secondly, such an analysis is beyond the scope of this review, which focuses on discussing the role of non-invasive fibrosis markers in detecting CVD risks in NAFLD patients. Hence, further research is needed to confirm the correlation between these diseases.

## 7. Conclusions 

Cardiovascular events are now considered the primary cause of death in NAFLD patients, which are significantly associated with NAFLD independent of recognized risk factors. It seems that the increase in cardiovascular risks parallels the progression of NAFLD. In addition to serum fibrosis biomarkers, many studies have explored the role of serum/urine oxidative stress markers in predicting cardiovascular risks. Unlike liver biopsy and imaging methods, non-invasive biomarkers have certain advantages in detecting cardiovascular risks due to the characteristics of being easily available, cheap, repeatable, and dynamic. Non-invasive serum tests are expected to be the first-line tools in CVD screening and risk stratification for NAFLD patients. Future research is needed to establish the corresponding cut-off values for specific CVDs and determine whether improved advanced fibrosis evaluated by fibrosis serum markers reduces cardiovascular risks.
